# Protocol and short-term results for a feasibility randomized controlled trial of a video intervention for Veterans with obesity: The TOTAL (Teaching Obesity Treatment Options to Adult Learners) pilot study

**DOI:** 10.1016/j.conctc.2021.100816

**Published:** 2021-06-29

**Authors:** Luke M. Funk, Catherine R. Breuer, Manasa Venkatesh, Anna Muraveva, Esra Alagoz, Bret M. Hanlon, Susan D. Raffa, Corrine I. Voils

**Affiliations:** a Department of Surgery, William S. Middleton VA, Madison, WI, USA; b Department of Surgery, University of Wisconsin-Madison, Madison, WI, USA; c Department of Biostatistics and Medical Informatics, University of Wisconsin-Madison, Madison, WI, USA; d Department of Veterans Affairs, National Center for Health Promotion and Disease Prevention, Durham, NC, USA; e Department of Psychiatry and Behavioral Sciences, Duke University School of Medicine, USA

**Keywords:** Behavior change, Obesity, Weight management medications, Bariatric surgery, Weight loss, Veterans health administration

## Abstract

**Introduction:**

All three evidence-based treatment options for adults with severe obesity – behavioral weight management, weight management medications (WMM), and bariatric surgery – are underutilized in the Veterans Health Administration (VHA) system. Our objective in this study was to develop and pilot-test the TOTAL (Teaching Obesity Treatment Options to Adult Learners) intervention, which seeks to increase Veteran participation in obesity treatment.

**Methods:**

In this single-site, parallel, pilot RCT, Veterans with severe obesity with an upcoming behavioral weight management visit were sent a recruitment letter after meeting inclusion/exclusion criteria via electronic health record screening. Eligible Veterans were randomized to TOTAL or usual care. TOTAL consisted of an 18-min video highlighting obesity health risks and treatment outcomes, eligibility criteria, and pros/cons of all three evidence-based obesity treatments. The primary outcomes were trial design feasibility (recruitment and retention rates) and acceptability to Veterans, which was assessed via semi-structured interviews with participants one week after randomization to TOTAL. Secondary outcomes included attitudes and self-efficacy to initiate treatment one week post-randomization and BMI change six months post-randomization (assessed via Cohen's d).

**Results:**

Forty-two Veterans were randomized (recruitment rate = 47.2%), and 40/42 completed one-week assessments (retention rate = 95.2%). The mean participant age was 59.2 ± 11.9 years. Female and non-White participants comprised 14.3% and 11.9% of the cohort, respectively. Semi-structured interviews with all 20 participants who received TOTAL suggested that the delivery logistics and content of TOTAL were acceptable to Veterans. Attitudes toward behavioral weight management and bariatric surgery and weight loss improved in TOTAL vs. usual care patients (Cohen's d ranging from 0.3 to 0.6).

**Conclusions:**

TOTAL was feasible to implement, acceptable to Veterans, and has the potential to increase obesity treatment participation in VHA. An adequately powered RCT is warranted to assess its impact on population-level weight loss.

**Trial registration:**

ClinicalTrials.gov NCT03856320.

## 1 Introduction

Severe obesity, defined as a body mass index [BMI] ≥ 35 kg/m^2^, affects nearly 20 million adults in the U.S [1]. Within the Veterans Health Administration (VHA) system, which is the largest integrated health care system in the U.S., nearly 800,000 Veterans meet BMI criteria for severe obesity [2]. Severe obesity is a highly morbid and costly chronic health condition. More than 70% of adults with severe obesity have “obesity-related” comorbidities, including cardiovascular disease, type 2 diabetes, obstructive sleep apnea, fatty liver disease, and certain cancers [[3], [4], [5], [6]]. Compared to adults with normal weight, individuals with severe obesity generate nearly $2000 more in annual medical costs [7], which suggests that Veterans with severe obesity generate more than $1 billion in excess medical expenditures each year.

All three evidence-based obesity treatments – behavioral weight management (including diet and physical activity), weight management medications (WMM), and bariatric surgery – are significantly underutilized in VHA. In fiscal year 2016, less than 10% of Veterans with obesity participated in VHA's behavioral weight management program (MOVE!) [8,9], which includes provider- or Veteran-initiated referrals for individual or group-based visits. Furthermore, only 7% of Veterans with obesity participate in more than one session annually [10], despite the VA clinical practice guideline recommending ≥12 visits annually [11]. Only 2% of Veterans who are eligible for WMM receive them [12]. Less than 0.1% of Veterans who meet BMI criteria for bariatric surgery undergo it annually at one of the 21 VHA bariatric surgery centers [9,13]. Three significant barriers to obesity treatment participation in VHA are poor Veteran knowledge about the risks of obesity, lack of awareness of obesity treatment options, and lack of Veteran motivation to participate in obesity treatment [12,[14], [15], [16], [17], [18]]. No existing interventions or adequately powered trials in VHA have sought to increase participation in all three obesity treatments.

The objective of this study was to develop and pilot-test a theoretically informed behavioral intervention (TOTAL: Teaching Obesity Treatment Options to Adults Learners) to increase obesity treatment participation and weight loss for Veterans with severe obesity. We sought to investigate if TOTAL would be feasible to implement at a single VA medical center (VAMC) and be acceptable to Veterans. Secondary outcomes included knowledge and attitudes toward obesity treatments, behavioral intentions, subsequent participation in obesity treatment, and weight loss.

## 2 Materials and methods

### 2.1 Theoretical approach and intervention design

**Fig. 1:**
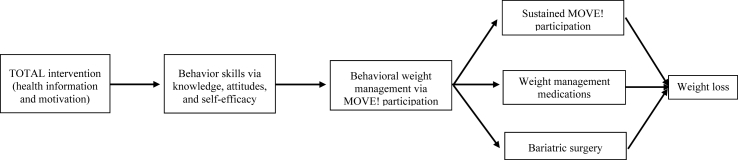
Conceptual model.

We applied Fisher's Information-Motivation-Behavioral Skills (IMB) conceptual model [19] as the theoretical foundation for TOTAL. The IMB model characterizes health-related information and motivation as contributors to behavior skills that affect health outcomes. Patients who are well informed, motivated to act, and have the required behavioral skills for effective action are more likely to initiate health-promoting behaviors – in this case, obesity treatment seeking [19]. Health-related information includes facts about health promotion and material addressing lay theories (e.g., “bariatric surgery is a dangerous procedure”). Motivation can be personal (e.g., attitudes toward weight loss treatment) and social (e.g., social support for seeking treatment) in nature. Behavioral skills include a patient's objective abilities to execute a behavior (e.g., initiate a discussion about starting WMM) and their self-efficacy to do so (e.g., does the Veteran believe he/she can obtain WMM). The TOTAL intervention comprises a video that imparts information and seeks to enhance motivation via improved knowledge, attitudes, and self-efficacy for pursuing any of the three evidence-based obesity treatments via MOVE! participation, which is required for WMM and/or bariatric surgery as well (Fig. 1).

To create the TOTAL video, we reviewed all codes from 73 interviews with Veterans with obesity and VHA providers. VHA providers included primary care providers [PCPs], bariatric surgeons, dietitians, and health psychologists. We have published the interview guides, study procedures and findings previously [18,20]. Themes that were pertinent to each IMB domain (e.g., Veteran motivations for achieving weight loss) were incorporated into a transcript that was written for the TOTAL video. To obtain feedback from a racially diverse sample of Veterans with severe obesity, we conducted four audio-recorded stakeholder engagement sessions with 34 Veterans in Chicago, IL (Jesse Brown VAMC) and Madison VAMC. Each group of Veterans watched the video and were asked open-ended questions about the content and audio-visual components of the video. Written summaries were generated by the study team after listening to the audio recordings. The video was modified after each stakeholder engagement session. The study PI subsequently conducted six interviews with providers (two PCPs, two bariatric surgeons, one dietitian, and one health psychologist) and obtained feedback from the VHA National Program Director for Weight Management (MOVE!). The study PI took notes and reviewed these notes with the study team. The video was modified one additional time after these interviews. Based on this comprehensive feedback, we modified the TOTAL video to include more information about MOVE! and WMM and revised some of the graphical displays and language to be more “user friendly.”

Eight behavior change techniques [21] were utilized to increase knowledge, improve attitudes toward each treatment, and increase self-efficacy for pursuing each treatment (Table 1). We sought to increase obesity-related health information by discussing the health consequences of obesity and providing instruction on how to seek weight management treatment. We aimed to enhance motivation by describing the pros and cons of each obesity treatment. In terms of behavioral skills, we incorporated Veteran testimonials to increase behavioral intention formation and self-efficacy via vicarious learning. We also attempted to create positive attitudes toward obesity treatment by providing information from a credible source (e.g., introduction by a VHA surgeon), providing information about the health consequences of each treatment (including pros and cons), and highlighting the salience of the consequences of not seeking treatment.

**Table 1:** TOTAL content for the pilot RCT.

Time interval	General topic	Video components	Behavior change technique
0:00–3:04	Obesity education and treatment option awareness	- Goals of the video (raising awareness of and motivation to pursue obesity treatment options) - Effect of obesity on Veterans, including health conditions - Explanation of BMI; examples of weights, heights, and BMIs - Description of three evidence-based obesity treatments in VHA	- Information about health consequences of seeking obesity treatment - Credible source
3:05–5:51	Behavioral weight management (MOVE!)	- Description of the MOVE! program, including lifestyle changes: diet and physical activity - Average expected weight loss with MOVE! - VHA eligibility criteria for MOVE! - Pros and cons of participating in MOVE!	- Instruction on how to participate in MOVE! - Health consequences of MOVE! participation - Salience of consequences of MOVE! participation - Pros/cons
5:52–9:24	Weight management medications	- Description of the types of FDA-approved medications and how they work - Average expected weight loss with obesity medications - VHA eligibility criteria for medications (including BMI criteria) - Pros and cons of taking obesity medications	- Instruction on how to pursue WMM - Health consequences of taking WMM - Salience of consequences of taking WMM - Pros/cons
9:25–17:37	Bariatric Surgery	- Description of bariatric surgery types (gastric bypass, sleeve) - Average expected weight loss with bariatric surgery - Common misconceptions about bariatric surgery - VHA eligibility criteria for bariatric surgery (including BMI criteria) - Pros and cons of undergoing bariatric surgery - Nutritional guidelines to follow after bariatric surgery - Veteran testimonials	- Instruction on how to pursue bariatric surgery - Health consequences of undergoing bariatric surgery - Salience of consequences - Pros/cons - Identity with the changed behavior - Vicarious learning - Information about other's approval
17:38–18:16	Summary	- Video summary and suggestion to contact MOVE! team if Veteran would like to pursue obesity treatment	- Instruction on how to pursue obesity treatment - Credible source

The final TOTAL video included approximately 3 min on obesity education and awareness of treatment options and 6 min on MOVE! and WMM (descriptions of the treatment components, average expected weight loss, VHA eligibility criteria, and pros and cons of each treatment). Given the added complexity of pursuing bariatric surgery and adhering to recommended post-operative care, nearly 8 min were focused on bariatric surgery-related information, including descriptions of the two most commonly performed bariatric procedures (sleeve gastrectomy and Roux-en-Y gastric bypass) and the required post-operative behaviors.

### 2.2 Study design

This pilot study is a randomized, parallel-design, single-site, two-arm trial. CONSORT guidelines for randomized pilot and feasibility trials were followed [22]. The study protocol was approved by the University of Wisconsin-Madison Institutional Review Board (IRB#: 2018–1216) and the Research and Development Committee at the Madison VAMC.

### 2.3 Setting and recruitment

The electronic health records (EHRs) of Veterans with an upcoming dietitian-led MOVE! visit between June 2019 and February 2020 at the Madison VAMC were screened to identify eligible study participants. Inclusion criteria at this stage included: age 18–75 who met BMI eligibility criteria for all three evidence-based obesity treatments (BMI≥35 kg/m^2^). Exclusion criteria at this stage included: active cancer diagnosis, pregnancy or stated intent to become pregnant, current breast-feeding, a severe psychiatric or substance use disorder precluding meaningful participation in the study, or previous bariatric surgery (or a referral within the past 12 months). Veterans meeting these criteria were mailed a recruitment letter, and those who responded were screened by phone. Exclusion criteria applied at this stage were lack of regular access to a telephone or non-English speaking.

Eligible and interested Veterans were scheduled for randomization on the day of their upcoming MOVE! visit. Recruitment ended when study enrollment exceeded our pre-determined sample size target. We attempted to over-recruit female and non-White participants to ensure that at least 30% and 20% of our cohort met these criteria, respectively. To operationalize this goal, we contacted every female and every non-White Veteran who met inclusion criteria and could be randomized by research staff on the day of the upcoming MOVE! visit.

### 2.4 Randomization and blinding

We randomized participants 1:1 to TOTAL or usual care. A random number generator was used to randomize participants in fixed block sizes of 8. All study team members were blinded to the block sizes except the biostatisticians (MV, BH). All participants arrived approximately 1 h prior to their scheduled MOVE! visit and signed an informed consent. The researcher then opened a sealed envelope to reveal intervention assignment. The researcher who randomized the participant was different from the researcher who assessed outcomes for the same participant.

### 2.5 Usual care arm

Participants in the usual care arm attended their MOVE! visit, which involved a 60-min individual or group visit with a dietitian. Following this MOVE! visit, participants may have chosen to either continue participation in MOVE! (by attending additional individual or group MOVE! visits) or discontinue MOVE! participation. Study participants also may have chosen to obtain information about WMM or bariatric surgery, either from a MOVE! provider or via discussions with other VHA providers.

### 2.6 Intervention (TOTAL) arm

Participants in the intervention arm watched the 18-min TOTAL video prior to their MOVE! visit. TOTAL was delivered in person by study personnel via an iPad and headphones. As with usual care, participants randomized to TOTAL may have chosen to continue participation in MOVE! (beyond their MOVE! visit on the day of randomized) and/or obtain information about WMM or bariatric surgery.

### 2.7 Study measures

We obtained age, sex/gender, race/ethnicity, education, marital status, employment, income, BMI, and MOVE! visit history via self-report during the baseline assessment. The presence of six obesity-related comorbidities (hypertension, type 2 diabetes mellitus, gastroesophageal reflux disease, obstructive sleep apnea, coronary artery disease, and dyslipidemia) during the past year was extracted from the EHR using ICD-10 codes. One-week and 6-month secondary outcomes were collected via telephone assessment and EHR review, respectively.

#### 2.7.1 Primary outcomes

The primary outcomes of this pilot RCT were focused on the feasibility of conducting a subsequent adequately powered RCT

*Feasibility:* We determined a priori that we would need to attain a 25% recruitment rate and 80% retention rate to enroll a sufficient number of participants into a subsequent adequately powered RCT. These criteria are commonly applied in weight management studies and have previously been used by our research group [23]. We defined the recruitment rate as the # of participants randomized/# of participants who were sent recruitment letters. We defined the retention rate as the # of participants who completed the one-week post-intervention assessments/# of participants randomized.

*Acceptability:* We assessed intervention acceptability via 15-min semi-structured interviews with all 20 participants who were randomized to TOTAL and completed the post-intervention assessment at one week. Participants were asked to discuss their opinions on optimal video delivery (e.g. in person or at home; group vs. individual MOVE! visit), video duration, and timing (e.g. before a MOVE! visit). These interviews were recorded, and written summaries were created by the two study team members who conducted the interviews. The written summaries were reviewed by the study team, and representative experiences and quotes were identified.

#### 2.7.2 Secondary outcomes

*One week outcomes* – Five psychologic constructs were assessed during the one-week post-randomization telephone assessment for all study participants. Participants were compensated $50 within one week of randomization.

1. *Preparation for decision-making:* Participants completed the Preparation for Decision-making questionnaire, which consisted of 10-items that assessed Veteran opinions about the effect of TOTAL on their decision-making process [24]. For example, “How much did your MOVE! visit help you recognize that a decision about weight management treatment needs to be made?” Response options ranged from 0 (not at all) to 4 (a great deal).
2. *Knowledge:* A 10-item questionnaire developed by Arterburn and colleagues was modified to assess knowledge and understanding of all three evidence-based obesity treatment options [25]. A mix of multiple choice and true/false questions were included. Total scores could range from 0 (least knowledge) to 10 (most knowledge).
3. *Attitudes:* Attitudes toward MOVE! WMM, and bariatric surgery were assessed via a 34-item questionnaire that used 7-point semantic differential scales (e.g., “I consider taking weight-management medications to be: beneficial/harmful, important/unimportant”). Item scores ranged from −3 (most negative attitude) to +3 (most positive attitude).
4. *Self-efficacy:* Self-efficacy to initiate any of the three evidence-based obesity treatments was assessed with items developed for this study following the methods of Schwarzer [26]. The 28 self-efficacy items began with the stem, “How sure are you that you can pursue (e.g., WMM) if”- and included endings such as “my weight loss is slower than I would like it to be.” Response options ranged from 1 (not at all sure) to 4 (very sure).
5. *Behavioral intentions:* Behavioral intentions to improve diet/physical activity, take WMM, or pursue bariatric surgery were assessed separately with five semantic differential items for each treatment. Response options ranged from 1 to 7 (unlikely to likely; impossible to possible; definitely would not to definitely would; no chance to certain; and probably not to probably) following the methods of Azjen [27].

*Six-month outcomes* – Four 6-month outcomes were assessed for all participants via EHR review.

1. *# of MOVE! visits:* We defined this as the # of dietitian-led MOVE! visits the study participant attended following randomization (not including the visit on the day of randomization).
2. *Weight management medication use (Yes/No):* We defined WMM use as the presence of an FDA-approved medication with criteria for use [CFU] for chronic weight management within VHA on the active medications tab in the EHR. Three of these medications are available on the VHA National Formulary (orlistat, phentermine/topiramate, and naltrexone/bupropion), while the fourth (liraglutide [SAXENDA]) is available non-formulary.
3. *Bariatric surgery referral (Yes/No):* We defined bariatric surgery referral as any referral to a bariatric surgery program within or outside VHA. Although receipt of bariatric surgery is the goal for Veterans who are referred, we reported referral for two reasons. First, it typically takes at least 6 months for Veterans to progress from a referral to surgery (in both VHA and non-VHA settings). Second, many factors contribute to whether a patient who is referred actually undergoes surgery. These include bariatric program-specific requirements such as alcohol and smoking cessation preoperatively, pre-operative risk evaluation for appropriateness for surgery, and mandatory pre-operative weight loss. Those factors were not targeted by our intervention.
4. *BMI change:* We calculated BMI change as the BMI indicated in the EHR six months after randomization – BMI indicated in the EHR at the time of recruitment.

### 2.8 Sample size and power considerations

Given that the primary goal of our pilot RCT was to assess acceptability and feasibility, a power calculation was not performed. Our a priori sample size was at least 40 participants, which we determined would be sufficient to evaluate study design feasibility and acceptability [28].

### 2.9 Analyses

For categorical variables, frequencies were reported. Continuous variables were reported via means and standard deviations. For ordinal and continuous outcome variables, the difference between TOTAL and usual care groups was calculated using the independent, two-sample Cohen's d together with the associated 95% confidence interval [29]. Cohen's d values of 0.20–0.49, 0.50–0.79, and ≥0.80 were characterized as small, medium, or large effect sizes, per Cohen's original description [30]. All analyses were conducted using Stata 15.0.

## 3 Results

### 3.1 Study enrollment

**Fig. 2:**
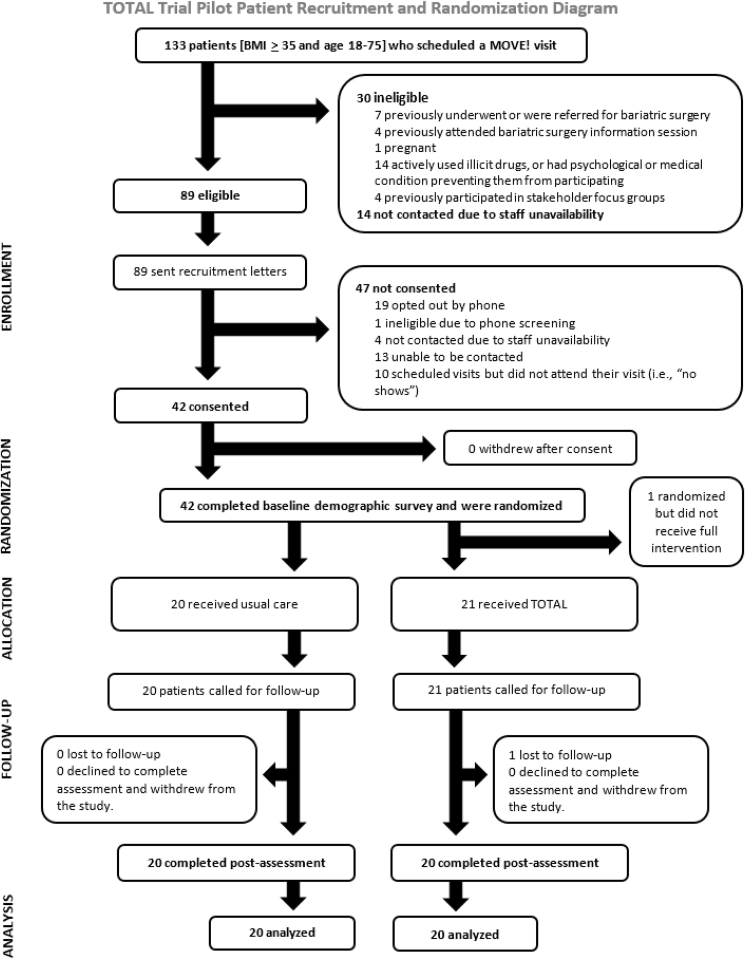
CONSORT flow diagram.

We identified 133 patients who met BMI and age criteria. Of those, 79.4% (n = 89) were eligible after chart review and were sent recruitment letters. The CONSORT flow diagram is shown in Fig. 2. Of the 89 eligible participants who were sent recruitment letters, 42 (n = 22 intervention, n = 20 usual care) were consented and were randomized (recruitment rate = 47.2%; Fig. 2). One participant who was randomized to receive TOTAL did not receive it because his MOVE! visit was canceled by clinic staff for reasons unrelated to the study. Another randomized to TOTAL was lost to follow-up. Thus, we reached 40 of 42 participants for the one-week follow-up telephone assessment (retention rate = 95%).

### 3.2 Participant characteristics

The mean age of our study participants was 59.2 ± 11.9 years. Approximately 14% were female, and 11.9% were non-White (Table 2). Nearly half (45.2%) were retired, and slightly less than half were employed. The average baseline BMI was 41.9 ± 6.6 kg/m^2^. All participants had at least one obesity-related comorbidity, and three-quarters had at least two obesity-related comorbidities.

**Table 2:** Participant characteristics.

Characteristic	Total (n = 42)	TOTAL (n = 22)	Usual care (n = 20)
Age Mean (SD)	59.2 (11.9)	60.7 (10.9)	57.5 (13.1)
Sex n (%)			
Male	35 (83.3%)	17 (77.3%)	18 (90%)
Female	7 (16.7%)	5 (22.7%)	2 (10%)
Gender n (%)			
Male	35 (83.3%)	17 (77.3%)	18 (90%)
Female	6 (14.3%)	5 (22.7%)	1 (5%)
Transgender	0	0	0
Other (e.g. preferred not to disclose)	1 (2.4%)	0	1 (5%)
Race/Ethnicity n (%)			
White, non-Hispanic	37 (88.1%)	19 (86.4%)	18 (90%)
Black, non-Hispanic	0	0	0
Hispanic	1 (2.4%)	1 (4.5%)	0
Other	4 (9.5%)	2 (9.1%)	2 (10%)
Marital Status n (%)			
Married/Partnered	25 (59.5%)	12 (54.5%)	13 (65%)
Divorced/Separated	13 (31%)	7 (31.8%)	6 (30%)
Single (never married or widowed)	4 (9.5%)	3 (13.6%)	1 (5%)
Education Level n (%)			
H.S. graduate or equivalent or less than H.S.	6 (14.3%)	4 (18.2%)	2 (10%)
Trade/technical/vocational school	3 (7.1%)	1 (4.5%)	2 (10%)
Associate's/Bachelor's degree, or some college	26 (61.9%)	13 (59.1%)	13 (65%)
Graduate/post-graduate work/degree (e.g., MA, PhD)	7 (16.7%)	3 (13.6%)	4 (20%)
Work Status n (%)			
Working part or full time	18 (42.9%)	10 (45.5%)	8 (40%)
Unemployed, searching for work	1 (2.4%)	0	1 (5%)
Retired	18 (42.9%)	10 (45.5%)	8 (40%)
Disabled	5 (11.9%)	2 (9.1%)	3 (15%)
Financial Status n (%)			
Have difficulty paying bills no matter what	1 (2.4%)	0	1 (5%)
Have enough to pay bills but have to cut back on things	4 (9.5%)	1 (4.5%)	3 (15%)
Have enough to pay bills but little to spare for special things	18 (42.9%)	9 (40.9%)	9 (45%)
After paying bills, I still have enough for special things	16 (38.1%)	9 (40.9%)	7 (35%)
I prefer not to answer	3 (7.1%)	2 (9.1%)	1 (5%)
BMI at time of recruitment Mean (SD)	41.9 (6.6)	43.1 (5.3)	40.7 (7.8)
Comorbidities n (%)			
Coronary artery disease (CAD)	9 (21.4%)	5 (22.7%)	4 (20%)
Gastroesophageal reflux disease (GERD)	21 (50%)	12 (54.5%)	9 (45%)
Hyperlipidemia	33 (78.6%)	19 (86.4%)	14 (70%)
Hypertension	30 (71.4%)	13 (59.1%)	17 (85%)
Obstructive sleep apnea	31 (73.8%)	16 (72.7%)	15 (75%)
Type II diabetes mellitus	21 (50%)	10 (45.5%)	11 (55%)
1–2 of the above comorbidities	10 (23.8%)	6 (27.3%)	4 (20%)
>2 of the above comorbidities	32 (76.2%)	16 (72.7%)	16 (80%)
Previous MOVE! visit participation n (%)			
Yes	39 (92.9%)	19 (86.4%)	20 (100%)
No	3 (7.1%)	3 (13.6%)	0
MOVE! visit type* n (%)			
Individual	20 (48.8%)	12 (57.1%)	8 (40%)
Group	21 (51.2%)	9 (42.9%)	12 (30%)

^+^One participant was randomized but did not attend the subsequent MOVE! visit.

### 3.3 Intervention acceptability

Interviews with the 20 participants who received TOTAL indicated that they were satisfied with its content, quality, and duration. Participants found the video to be “very informative,” “well presented,” and “easy to understand.” One stated the video “sparked [his] interest” in weight loss options beyond dietary changes and physical activity and prompted him to initiate a conversation about WMM and bariatric surgery with the MOVE! dietitian. Most Veterans said the video duration was “just right.” Most found it helpful to watch the video before their MOVE! visit because it allowed them to ask directed questions during the ensuing MOVE! visit. Opinions regarding whether the video should be watched in a clinic setting or home/online were divided.

### 3.4 One-week outcomes

The means, standard deviations, and effect sizes (Cohen's d) for between-group differences in one-week outcomes are shown in Table 3. Knowledge was high for both groups (8.3 ± 0.8 for usual care vs. 8.5 ± 1.3 for the TOTAL group). In both groups, attitudes and behavioral intentions were more favorable/stronger for MOVE! compared to WMM and bariatric surgery. The effect sizes ranged from 0.01 (self-efficacy for weight management medications and bariatric surgery and behavioral intentions for MOVE! and bariatric surgery) to 0.6 (preparation for decision-making). All 95% confidence intervals included 0.

**Table 3:** Secondary outcomes.

Outcome	Usual care (n = 20)	TOTAL (n = 20)	Cohen's d (95% CI)
Psychologic outcomes assessed at one-week (means, SD)^a^			
Preparation for decision-making	2.8 (0.7)	3.1 (0.5)	0.6 (−0.01, 1.3)
Knowledge of treatment options	8.3 (0.8)	8.5 (1.3)	0.2 (−0.4, 0.8)
Attitudes (−3 to 3)			
MOVE!	2.0 (0.9)	2.4 (0.7)	0.5 (−0.1, 1.1)
Weight management medications	0.7 (1.4)	0.8 (1.5)	0.1 (−0.5, 0.7)
Bariatric surgery	−0.3 (1.6)	0.1 (1.9)	0.3 (−0.3, 0.9)
Self-efficacy (0–4)			
MOVE!	2.8 (0.6)	2.8 (0.5)	.04 (−0.5, 0.7)
Weight management medications	2.7 (0.9)	2.7 (0.7)	.01 (−0.6, 0.6)
Bariatric surgery	2.7 (1.0)	2.6 (0.8)	.01 (−0.5, 0.7)
Behavioral intentions (0–7)			
MOVE!	6.1 (0.8)	6.0 (0.8)	.01 (−0.6, 0.7)
Weight management medications	3.7 (2.6)	3.9 (2.0)	0.1 (−0.5, 0.7)
Bariatric surgery	2.4 (1.9)	3.1 (2.1)	.01 (−0.05, 0.7)
**Utilization and weight loss assessed at 6 months**			
# MOVE! visits (median, IQR)	3 (1.5, 6.5)	1.5 (0, 4.5)	
Weight management medication use (n, %)	2 (10%)	5 (25%)	
Bariatric surgery referral (n, %)	0	1 (5%)	
BMI change (kg/m^2^, SD)	−0.1 (2.0)	−0.8 (1.2)	0.4 (−0.2, 1.0)

a Preparation for decision-making response options ranged from 0 (not at all prepared) to 4 (maximally prepared). Knowledge scores could range from 0 (least knowledge) to 10 (most knowledge). Attitude response options ranged from −3 (most negative attitude) to +3 (most positive attitude). Self-efficacy response options ranged from 0 (not at all sure) to 4 (very sure). Behavioral intention response options ranged from 0 (lowest intention) to 7 (strongest intention).

### 3.5 Six-month outcomes

Over six months of follow-up, the median number of MOVE! visits was 3 (IQR 1.5, 6.5) for the usual care group and 1.5 (IQR 0–4.5) for the TOTAL group. Two usual care participants (10%) received a new WMM or bariatric surgery referral compared to 6 (30%) TOTAL patients. The effect size for BMI change was 0.4 (95% CI -0.2, 1.0). The TOTAL group BMI change was −0.8 kg/m^2^ ± 1.2) compared to −0.1 ± 2.0 for the usual care group).

## 4 Discussion

Our pilot RCT findings suggest that TOTAL was feasible to implement and acceptable to Veterans. Our qualitative interview findings suggested that Veterans felt they were better informed about available obesity treatment options in VHA. Effect size estimates for knowledge, attitudes, and BMI suggested that TOTAL may have improved these outcomes. However, the 95% CIs all included 0, which prohibits any definitive conclusions regarding these outcomes. Several “lessons learned” will impact the design and execution of a subsequent adequately powered efficacy RCT proposal.

First, both TOTAL and usual care participants had higher than expected knowledge about obesity and its treatment options. This left little room for improvement among participants who received TOTAL. This may have been due to the severity of their obesity, which may have prompted more frequent obesity-related discussions with VHA providers, as suggested by their previously scheduled MOVE! visit. Our proposal for an adequately powered trial will target Veterans who have not participated in MOVE! within the past year and are likely less knowledgeable about obesity treatment options. Furthermore, we will expand our inclusion criteria to target Veterans who meet BMI criteria for MOVE! and WMM (BMI≥27), but may not meet criteria for bariatric surgery (BMI≥35). We expect that knowledge about obesity and its health risks will be lower in this cohort given that fewer will have severe obesity and the comorbidities that prompt weight-related discussions with providers, such as managing hypertension or type 2 diabetes [31], Veterans who learn about bariatric surgery but do not meet BMI criteria may still benefit from learning about it now as a treatment to pursue (or avoid) if they meet BMI criteria in the future.

Second, the impact of TOTAL could be expanded if the subsequent efficacy trial targeted a broader group of Veterans with obesity. Nearly 90% of Veterans (approximately 3.5 million Veterans) who meet BMI eligibility criteria for MOVE! do not participate in it [8,9]. More than 98% who meet BMI eligibility criteria for WMM do not receive them [12]. To maximize the impact of TOTAL, we intend to target Veterans who are not actively participating in MOVE! and are not receiving WMM. As indicated in the Fisher conceptual model, motivation is a critical determinant of whether behavior will change [19]. Since Veterans not participating in MOVE! will, by definition, be less motivated compared to our pilot study participants, the motivational component of TOTAL will be enhanced by incorporating motivational telemedicine calls. These telemedicine sessions will contain motivational elements, such as encouraging Veterans to focus on the problem to change, evoking their desire to change, and helping them plan that change [32].

Third, a single-site trial at the Madison VAMC will not be sufficient for obtaining an adequately diverse study population given that we failed to meet our female sex and race/ethnicity enrollment targets. To achieve more ethnic/racial and geographic diversity, we will expand our study proposal to multiple VAMCs, including those with a higher proportion of non-White Veterans. If a multi-site, adequately powered RCT shows that TOTAL is efficacious, we will seek to disseminate TOTAL throughout the VA system. The potential impact of TOTAL could be significant given that all Veterans who meet BMI criteria have access to all three evidence-based treatments. Per VA directive, MOVE! is required to be available to Veterans at all 140 VAMCs [33]. Multiple obesity medications are on the VA formulary that is used by providers at all facilities [12]. Thus, all Veterans who meet criteria for use could be prescribed these medications. All 18 Veteran Integrated Service Networks (i.e., sub-regions throughout the country that all VAMCs are a part of) have designated bariatric surgery referral centers within VA [13]. Although bariatric surgery may not be offered at a Veteran's main facility, Veterans can travel to another facility within their VISN to receive surgery.

Although the effect sizes for knowledge, attitudes, and BMI change suggested that TOTAL may have improved these outcomes, the 95% CIs for all of the measures included 0. This may have been due to the intentionally limited sample size in our feasibility pilot. It is also possible that TOTAL did not affect these constructs and instead drove behavior via other behavioral constructs. Both WMM and bariatric surgery utilization were slightly higher in the TOTAL group, while the median number of MOVE! utilization was higher in the usual care group. Although statistical testing of the number of MOVE! visits was not performed due to the limited sample size, this latter finding may have been due to several outliers of high MOVE! participation in the usual care group. The qualitative feedback we received from TOTAL participants during post-intervention interviews suggested that TOTAL made participants more likely to want to participate in MOVE! Attitudes were more favorable toward MOVE! and behavioral intentions were more positive toward MOVE! in both groups, compared to WMM and bariatric surgery. These are potential targets for modification of TOTAL prior to a subsequent multi-site trial.

There are several limitations to this feasibility pilot. First, eligible participants were identified by manual review of clinic schedules in the EHR. This was feasible in this single site pilot study, but will not be feasible in a larger efficacy trial that enrolls several hundred patients from multiple sites. To address this concern, we performed a post-hoc electronic data pull using national data from the VA corporate data warehouse (CDW), which is the approach we will apply in a subsequent trial. Every participant who met age and BMI criteria was identified via the electronic data pull, which suggests this approach is highly sensitive for identifying eligible Veterans. Second, our study participants were mostly male given that the VHA patient population is primarily male. Our findings may not be generalizable to a non-Veteran population seeking obesity treatment. Third, the TOTAL intervention was delivered in person, which may not be feasible for dissemination and implementation nationally throughout VA system. We are currently pilot-testing administration of TOTAL via VA Video Connect, which is a telemedicine platform supported by VA. Finally, TOTAL was delivered to Veterans who were already motivated enough to have a MOVE! visit scheduled. It is unclear if TOTAL would be effective if it were offered to Veterans who are not actively participating in MOVE!

In summary, with this pilot RCT work completed, we are poised to investigate whether our theoretically grounded behavioral intervention is efficacious at increasing obesity treatment participation and weight loss for Veterans. Given that overweight/obesity affects nearly 8 in 10 Veterans, TOTAL has potential for significant impact in VHA. If effective, TOTAL could be incorporated into existing VHA operations and clinical infrastructure, including MOVE! to support rapid implementation and subsequent weight loss for Veterans with obesity.

## Declaration of competing interest

The authors declare that they have no known competing financial interests or personal relationships that could have appeared to influence the work reported in this paper.
